# Evidence for the *DRD2* Gene as a Determinant of Reward Deficiency Syndrome (RDS)

**Published:** 2023-06-29

**Authors:** Kenneth Blum, Abdala Bowirrat, Igor Elman, David Baron, Panayotis K. Thanos, Mark S Gold, Colin Hanna, Milan T. Makale, Keerthy Sunder, Nicole Jafari, Foojan Zeine, Kevin T. Murphy, Miles Makale, Rajendra D. Badgaiyan

**Affiliations:** 1 Department of Molecular Biology, Adelson School of Medicine, Ariel University, Ariel, Israel; 2 Division of Addiction Research & Education, Center for Exercise Sports, Mental Health, Western University Health Sciences, Pomona, CA, USA; 3 Institute of Psychology, Eötvös Loránd University Budapest, Hungary; 4 Department of Psychiatry, Boonshoft School of Medicine, Wright University. Dayton, OH., USA; 5 Department of Psychiatry, Human Integrated Services Unit, University of Vermont Center for Clinical & Translational Science, College of Medicine, Burlington, VT, USA; 6 Department of Psychiatry, Harvard College of Medicne, Cambridge, MA., USA; 7 Behavioral Neuropharmacology and Neuroimaging Laboratory on Addictions, Clinical Research Institute on Addictions, Department of Pharmacology and Toxicology, Jacobs School of Medicine and Biosciences, State University of New York at Buffalo, Buffalo, NY, USA; 8 Department of Psychiatry, Washington University School of Medicine, St. Louis, MO 63110, USA; 9 Department of Radiation Medicine and Applied Sciences, University of California San Diego, La Jolla, CA., USA; 10 Department of Psychiatry, University California, UC Riverside School of Medicine, Riverside, CA., USA; 11 Division of Neuromodulation Research, Karma Doctors & Karma TMS, Palm Springs, CA., USA; 12 Sunder Foundation, Palm Springs, CA., USA; 13 Department of Human Development, California State University at Long Beach, Long Beach, CA., USA,; 14 Division of Personalized Medicine, Cross-Cultural Research & Educational Institute, San Clemente, CA., USA. 15Awareness; 15 Integration Institute, San Clemente, CA., USA; 16 Department of Health Science, California State University at Long Beach, Long Beach, CA. USA; 17 Division of Personalized Interventions, Peak Logic, Del Mar CA., USA; 18 Department of Psychology, University of California San Diego, La Jolla, CA., USA; 19 Department of Psychiatry, Long School of Medicine, University of Texas, Health Science Center, San Antonio, Tx., USA

**Keywords:** GWAS, Candidate genes, *DRD2* gene, Polymorphisms, Hypodopaminergia, Reward deficiency and addictive and non- addictive behaviors

## Abstract

Since 1990, published addiction psychiatry articles have exceeded 11,495. Several from Blum et al. showed the clinical relevance of the Genetic Addiction Risk Severity (GARS) test in identifying risk for reward deficiency behaviors in cohorts from polysubstance and pain clinics, post-surgical bariatrics, and DWI offenders facing prison time. Since Blum *et al* first published in JAMA (1990) concerning the association of the *DRD2* gene polymorphism and severe alcoholism, confirmation has been mixed and controversial. More recently, however, a meta-analysis of 62 studies showed a significant association between *DRD2* rs 1800497 and Alcohol Use Disorder (AUD). Other studies from Yale University showed that a haplotype block of the *DRD2* gene A1 allele was associated with AUD and heroin dependence. GWAS studies of depression and suicide in 1.2 million veterans confirmed the first psychiatric candidate gene study finding from Blum et al. 1990; a significant association between the minor *DRD2* allele, Taq A1 (rs 1800497 C>T) and severe alcoholism. Additionally, the *DRD2* rs1800497 is associated with suicide behaviors robustly at P=1.77 × 10^−7^. Furthermore, DNA polymorphic alleles underlying SUD with multiple substances were mapped via chromatin refolding, revealed that the *DRD2* gene and associated polymorphism(s) was the top gene signal (DRD2, P=7.9 × 10^−12^). Additionally, based on these investigations, we conclude that GWAS should end the controversy about the *DRD2* gene being at least one determinant of Reward Deficiency Syndrome (RDS) first reported in the Royal Society of Medicine journaling 1996.

## Introduction

### Early evidence and controversy

In 1990, Kenneth Blum, Ernest Noble, and their esteemed colleagues set the world on its heels with the publication of their seminal research on the first-ever discovery of a gene polymorphism associated with Severe Alcoholism [1,2]. Specifically, in a blind experiment, they reported the first allelic association of the dopamine D2 receptor gene in alcoholism. The presence of the A1 allele of the dopamine D2 receptor gene correctly classified 77% of subjects known to have AUD, and its absence classified 72% of subjects without AUD. The polymorphic pattern of this receptor gene suggests that susceptibility to at least one form of alcoholism is conferred by a gene located on the q22-q23 region of chromosome 11. Blum and Noble pointed out that the *DRD2* A1 allele was not specific for alcoholism but was a reward-linked genetic polymorphism. Coverage of these findings through the JAMA media network was extensive, included every news media outlet worldwide, and garnished the excitement of the entire addiction psychiatry community. “Discovery Magazine” honored the work by its inclusion in its prestigious 25 most important discoveries of 1990. In fact, before this finding, a Gallop Poll in America reported that only a minority of the populace believed that alcoholism was a neurobiological disorder having genetic underpinnings. However, shortly following this JAMA report, a newer Gallop Poll found that the popular opinion shifted, whereby most American polled now believed in a plausible genetic basis of alcoholism. While the scientists at NIDA excitedly embraced this novel finding by Blum et al [1]. Unfortunately, the opposite was true for NIAAA scientists. An editorial from NIAAA scientists revealed that while they were excited about these findings, they questioned the validity because of such a robust finding with only one candidate gene polymorphism [3], and others, accompanying the original JAMA report [1]. This NIAAA editorial initiated the long-standing controversy over the role of the *DRD2 Taq A1* allele and its putative association with alcoholism. Along these lines, in 1990, Bolos and others from the section on Genetic Studies, NIAAA [4], comparing alcoholics (not all severe) with poorly screened controls, did not support a widespread or consistent association between the D2 receptor gene and alcoholism. Moreover, in a flawed study, Gelernter’s group [5], from Yale also did not find support for the *DRD2 TaqA1* allele and alcoholism. Reappraisal [6,7], and careful review revealed two important caveats which help explain the lack of association

- The control population was derived from a French Tourette’s Cohort
- Subjects with elevated SGOT liver enzyme levels were excluded (leaving not very severe alcoholics).

During this earlier period, there were positive associations with not only alcoholism but other addictions [8–14]. Moreover, Blum and Noble’s group [15], also found evidence for carriers of the *DRD2* A1 allele independent of alcoholism progressively reduced Bmax in subjects with A2/A2, A1/A2, and A1/A1 alleles, with subjects with A2/A2 having the highest mean values, and subjects with A1/A1, the lowest (up to 40% reduction) and confirmed by others [16]. Despite positive reports linking the *DRD2* gene Taq A1 allele to some reward deficit behaviors, the controversy gained steam with negative thinking by not only scientists but science reporters [17, 18]. The then director of the NIMH, Elliot Gershon, published an article in Science Magazine showing a shattered glass castle, suggesting that Blum and Noble live in a glass castle. Interestingly, dogmatic naysayers like David Goldman became less abrasive, and by 2019, his group reported applying a meta-analysis involving 62 studies of *DRD2* and AUD with 16, 294 participants. The rs1800497 SNP was associated with AUD (odds ratio, 1.23; 95% CI, 1.14–1.31; P<.001). They correctly pointed out that the association was attributable to spuriously low allele frequencies in controls in positive studies [19]. Although initially, this appears to be a negative comment, it is positive for this study. We have consistently argued that fewer reward deficiency behaviors in controls (thus the lower frequency of *DRD2* A1 and other alleles that induce functional hypodopaminergia) is mandatory, expected, and desirable in the accurate candidate as well as GWAS genetic investigations [20]. Reward Deficiency Syndrome (RDS) was coined by Blum et al. in 1995 to characterize a group of behaviors associated with the relative failure of the dopaminergic system, which plays a significant part in brain reward mechanisms linked to dopaminergic dysfunction; acute excess or chronic deficit of dopamine release in the brain reward circuitry. The reward deficiency behaviors include drug and non-drug addictive, compulsive, and impulsive behaviors [21, 22]. In 1995, Blum et al. [21], reported that based on a Bayes’ theorem calculation, the Predictive Value whereby carriers of the *DRD2* A1 allele would have a 74.4% chance of expressing any one of several RDS behaviors, both substance (like cocaine) or non-substance addictive behaviors (like gaming and pathological gambling). It is noteworthy, that the Journal of the Royal Society of Medicine published the first-ever peer-reviewed article concerning RDS. That article [22], proposed that dysregulation of the D2 dopamine receptors leads to aberrant substance-seeking behavior (alcohol, drug, tobacco, and food) and non-substance behaviors (pathological gambling, Tourette’s syndrome, Attention-Deficit Hyperactivity Disorder [ADHD]). We further proposed that specific D2 dopamine receptor gene polymorphisms are important common genetic determinants of reward deficiency. Currently, there are 1506 articles listed in PUBMED using the word search “ reward deficiency” and 244 articles specifically using the word search “reward deficiency syndrome” as of 6-15-2023. The finding of Neville et al. that the *DRD2* Taq1A RFLP is a SNP that causes an amino acid substitution within the 11^th^ ankyrin repeat of ANKK1 (p.Glu713Lys) helped us understand an alternative explanation for previously described associations between the *DRD2* Taq1A RFLP and neuropsychiatric disorders such as addiction [23]. In addition, Gelernter’s group also reported that for both AUD and drug dependence [24], the ANKK1 exon 8 to DRD2; C957T was significantly associated (p=0.0028) in both samples. Unfortunately, previously, the poor screening of controls (the non-removal of hidden RDS behaviors like obesity, ADHD, PTSD, and gambling may have produced a series of spurious results in gene-based investigations of the role of *DRD2* gene polymorphisms and neuropsychiatric disorders. These conflicting results called for “super controls” in the research [25]. The idea that the *DRD2* polymorphisms are overrepresented in childhood aggression has been confirmed by Zai et al., [26], from the Neurogenetics Section, Centre for Addiction and Mental Health, Toronto, Canada. Specifically, they reported that aggressive children compared to controls were significantly more likely to have at least one copy of the G allele for the *DRD2* A-241G polymorphism (genotypic P=0.02; allelic P=0.01); *DRD2* rs1079598 CC genotype (genotype P=0.04). The *DRD2* TaqIA T allele (P=0.01) was also significantly overrepresented in aggressive children. The *DRD2* gene is one of the most studied reward genes in psychiatry. Moreover, using the word search “Dopamine D2 Receptor there are 27,054 articles listed. Very early concepts, before the actual identification of the *DRD2* gene polymorphism [27], in a highly cited article in addiction psychiatry (1985), Dackis and Gold [28], suggested that dopamine depletion is the result of chronic cocaine abuse, and this depletion is the basis of the dysphoric effects of cocaine abstinence and cocaine urges. Notably, in comparison to the DRD1 gene, which is considered a driver at the VTA-glutaminergic construct (“GO”), the *DRD2* gene is the opposite and considered a filter or an inhibitory (“no Go”) gene that helps regulate dopamine activity [29].

## Literature Review

### Gwas-based clinical analytics supporting the formidable role of the *DRD2* gene polymorphisms in addiction psychiatry

**Figure 1.**
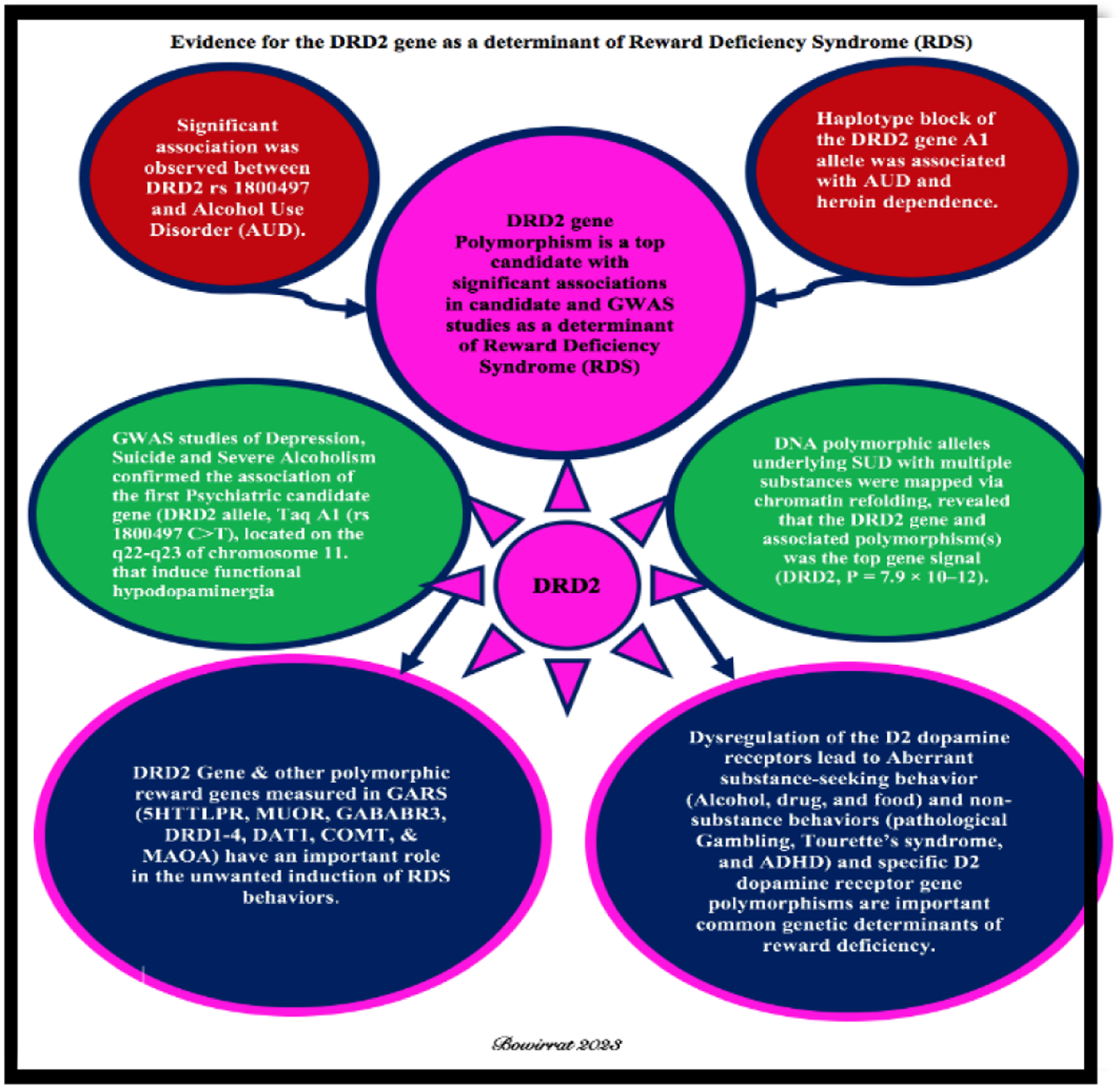
Evidence for the DRD2 gene as a determinant of Reward Deficiency Syndrome (RDS).

As mentioned earlier, the first-ever confirmed candidate psychiatric genetic association study performed by Kenneth Blum and Ernest Noble and their team discovered a significant association between the minor *DRD2* allele Taq A1 (rs 1800497 C>T) and severe alcoholism published in JAMA (1990) [1]. Moreover, a PUBMED search using “dopamine genes “revealed 9,643 articles as of 6-15-23 mainly related to addiction psychiatry. Since 1990, our laboratory has published many studies; positive clinically relevant analyses of utilizing GARS to help identify AUD (N=393) [30]; SUD (N=110,241 cases and 122,525 controls; high opioid risk in pain clinics (121 chronic opioid users) eating expectancy and other RDS behaviors in a bariatric post-surgical cohort (N=34) post clinical outcome one year follow-up [31–34]. Our group also utilized in drug Court to help convert DWI offenders facing prison time to rehabilitation based on genetic determinism rather than free will (N=31) saving over 225 years of prison time to mandated probation [35]. In 2014, Blum’s group developed the GARS test of ten genes and eleven SNPs across the finite neurotransmitter pathways representative of the Brain Reward Circuitry DNA hypodopaminergia antecedents to addiction psychiatry [31]. In 2022, involving 74,566 case-controls (AUD), Blum’s group statistically validated the selection of these risk alleles measured by GARS and showed significance for *DRD2, DRD3, DRD4, DAT1, COMT, OPRM1*, and *5HTT* at 5%. These alleles captured post-risk for 8% estimation of the population’s alcoholism prevalence post-risk for 8% estimation of the population’s alcoholism prevalence [36]. They genotyped over 3,000 people presenting with polysubstance abuse from at least one-dozen chemical dependency and Behavioral addiction clinics, including general population mixed gender and race that resulted in a GARS score for drug and alcohol risk at over 90% and 72% respectively [35,36]. While the initial discovery of the candidate association study of the *DRD2* A1 allele met with significant controversy over many years now this gene and associated polymorphisms have been confirmed in several elaborate GWAS investigation. Levey et al. from Yale, reported on a large meta-analysis of depression that used data from 23 and Me, the Million Veteran Program [37, 38], the UK Biobank, the FinnGen biobank, and subjects of European ancestry (n=1,154,267; 340,591) and African ancestry (n=59,600; 25,843). Remarkably, transcriptome-wide association study analyses showed significant associations with the expression of*NEGR1* (a dopamine regulatory gene) in the hypothalamus and *DRD2* in the nucleus accumbens, among others. This extensive investigation underscored the genetic architecture of depression and provided new insight into the interrelatedness of complex psychiatric traits. Another similar work by Kimbrel et al. from Duke [39], carried out a GWAS that identified gene polymorphic associations in pan-ancestry and ancestry-specific loci with attempted suicide among veterans. Once again, they found a robust pan-ancestry signal at the *DRD2* locus (p=1.77×10^−7^). Moreover, it was also identified and subsequently replicated in a large, independent international civilian cohort (p = 7.97 × 10^−4^). Of further interest, ancestry-specific genome-wide significant loci were also identified in African Americans, European-Americans, Asian Americans, and Hispanic Americans. In addition, pathway analyses yielded an impressive list of reward gene polymorphisms with high clinical significance, including glutamatergic synapse, dopaminergic synapse, oxytocin signaling, cortisol synthesis and secretion, and circadian rhythm. Most importantly, the authors suggest that their pathway analyses suggest that many commonly impacted biological pathways could inform the development of beneficial therapeutics for suicide prevention. The findings related to an expected genetic rubric load onto the RDS construct [40]. Finally, along similar lines, a new study confirms the RDS construct originally suggested by Blum’s group in 1995, and 1996 [21, 22]. It suggests that a common genetic signature of RDS may augment a person’s risk of developing substance use disorders, regardless of the substance of choice. The research from Washington University School of Medicine in St. Louis [2], as the authors pointed out, could eventually lead to universal therapies to treat multiple substance use disorders. Accordingly, the authors further point out that multiple genes link to regulating dopamine signaling within the genetic signature of their cohort associated with SUD. The authors correctly espouse that repeated exposures to addictive substances can result in adaptation in the dopamine pathway to the effects of these substances requiring more of the substance to receive the same reward (tolerance and craving). Hatoum et al., observed polymorphic alleles of DNA that underlie SUD with multiple substances [2], including 19 SNPs associated with general addiction risk and 47 DNA variants linked to the specific drug of abuse: 32 for tobacco, nine for alcohol, five for cannabis, and one for opioids. Importantly, their analyses highlight that the regulation or modulation of dopaminergic gene function, rather than just the presence of variation in dopaminergic genes, is central to general addiction liability. To help end the three-decade controversy, the *DRD2* gene and associated polymorphism were the top gene signal (DRD2 (P = 7.9 × 10^−12^), mapped via chromatin refolding. This finding suggested a regulatory mechanism for the *DRD2* gene (a “no go”). While they indicate that the role of striatal dopamine in positive drug reinforcement is well established [41], they failed to acknowledge earlier and recent work from Blum’s laboratory and associates related to the *DRD2* gene association with any addiction, [8, 13, 15, 25, 30, 31, 35, 36, 40], as well as over 200 more citations. Nevertheless, we applaud this work highlighting the importance of the shared genetic basis of addictions and the novel role of the interplay of reward and cognition, especially as it relates to impairments of dopaminergic function and regulation as an underlying cause of addiction with, of course, triggering epigenetic insults in general RDS [37]. In summary, these results over three decades indicate that not only the *DRD2* gene but other polymorphic reward genes measured in GARS (*5HTTLPR, MUOR, GABABR3, DRD1–4, DAT1*, *COMT*, AND *MAOA*), for example, have an important role in the unwanted induction of RDS behaviors [42–44]. In addition, it is genuinely interesting that gene therapy, as first studied by Panayotis Thanos and Nora D Volkow and others, consistently revealed that increasing the *DRD2* receptors via an adenoviral vector delivered into the nucleus accumbens of rats significantly reduced alcohol intake as well as cocaine self-administration [45–47]. It is well-known THAR should be that eating disorders and SUD frequently co-occur as described in the RDS construct. Research from the laboratory of Munn-Chernoff, Brown and others, observed that four eating disorder phenotypes (e.g., Anorexia Nervosa [AN], AN with binge eating, AN without binge eating, and a bulimia nervosa factor score) and eight SUD related phenotypes employing GWAS in up to 537 000 subjects, shared common genetic variants. Significant positive genetic associations emerged between AUD and AN (r_g_ =0.18; false discovery rate q = 0.0006) [lost significance after correcting with removal of major depression], cannabis initiation and AN (r_g_ = 0.23; q<0.0001), and cannabis initiation and AN with binge eating (rg= 0.27; q= 0.0016).The authors suggested that association between eating disorder- and substance-use-linked phenotypes reveal complex genetic interaction. In agreement with this important GWAS data, we further suggest that these results support the common genetic rubric of RDS. While we encourage the scientific community to perform additional required, more extensive studies (GWAS and Candidate approaches), we believe that GARS is a tool that can be used to identify addiction risk early in life, not to label but inform parents of potential danger ahead (Figure1).

## Conclusion

The consistent finding during recent years that the *DRD2* gene is a top candidate with significant associations in candidate and GWAS studies involving millions of people presenting with depression, suicide ideation, and attempted suicide, as well as SUD, provides enough evidence to satisfy the scientific community to disembark from the previous controversy related to whether this gene and associated polymorphisms are indeed linked to most if not all substance and non-substance behavioral addictions. In my opinion, if Noble were still alive, he would agree that the jury has spoken. So with this evidence we are proving that indeed the *DRD2* gene and associated polymorphisms are major DNA antecedents linked to “preaddiction” and should “not be” ignored but considered “ to be’ important.
